# Supplementary material for: Understanding factors responsible for the slow decline of soil-transmitted helminthiasis following seven rounds of annual mass drug administration (2012–2018) among school children in endemic counties of Kenya: A mixed method study
Source: PLoS Negl Trop Dis. 2023 May 4;17(5):e0011310. doi: 10.1371/journal.pntd.0011310 (PMC10187929; doi:10.1371/journal.pntd.0011310)
Supplement: Attachment — Submitted filename: Response to reviwers.docx [file pntd.0011310.s001.docx]

The Chief Editor,

PLOS Neglected Tropical Diseases,

Dear Sir/Madam,

**REFERENCE: SUBMISSION OF A REVISED MANUSCRIPT**

We are hereby, submitting a revised manuscript titled **“Understanding factors responsible for the slow decline of soil-transmitted helminths following seven rounds of annual mass drug administration (2012 – 2018) among school age children in endemic counties of Kenya: A mixed method study”**, for your consideration for publication in your esteemed journal.

We have acted on the reviewer’s comments as listed below;

Reviewer #1: - The objectives of the study can be discerned from the background section but could be more clearly presented. A testable hypothesis was not provided.

The hypothesis testing statement has been included; line 143-145: H_0_ there were no factors associated with the slow decline in prevalence and intensity of STH among SAC participated in the school deworming programme.
- The choice of cross-sectional study is appropriate to the subject. However, the design does not seem to have been developed based on a clear conceptual framework of the transmission of STH and the links with WASH. This should ideally be presented in the background/introduction section, including the following elements:
* Set out the mode of transmission of STH, explaining clearly the need for egg maturation in the soil and the difference with hookworm - this is crucial as it affects the importance of different elements of WASH to each of the STHs. It would also be useful to refer to the resilience of helminth eggs in the environment at this stage (only alluded to much later in the paper) – Introduction this is crucial since it can cause significant time lags between improvements in sanitation, and reduced prevalence, which can only be achieved through reducing the overall contamination of the environment. This has been addressed see line 75-77; line 133-135.

A conceptual framework has been done see line 149
* Provide a definition of WASH (noting that this concept includes a vast array of interventions and outcomes, but it is being used in a monolithic way throughout the paper).

WASH has been defined in line 83.

The authors are advised to revisit the WHO Guidelines on Sanitation and Health for a more detailed examination of the sanitation service chain and its relationship with pathogen transmission, since toilet presence and open defecation are only two potential factors.

This has been addressed in line 116-131.
The authors seem to have made certain assumptions about the routes of transmission, which has left out important aspects such as food crop contamination. This has also made it difficult for the authors to make clear links between the behaviours and characteristics included in the study, and the risk of STH transmission. For instance, while handwashing with soap after toilet use may be a useful proxy indicator of handwashing practices, it is less likely to prevent infection than, for instance, handwashing before eating (since the infective eggs on hands are more likely to come from the environment than from contaminated toilet surfaces, as the eggs there are unlikely to have matured). Similarly, while toilet presence in the household is a useful indicator of open defecation practices, toilet type, or whether or not the toilet is considered improved or not, is an important indicator of the risk of contamination of the environment, and this was not included.
At household level the type of latrine was not measured since the children were reporting this measurements while in school and they would not have sufficiently known whether the household latrine is improved or not
The authors relied on questionnaires and focus group discussions. It is unclear what efforts were made to triangulate or verify the information gathered. Observations of school sanitation facilities are mentioned in the discussion section, but no observations are detailed in the methods section, or any protocol that may have been used to undertake such observations in a structured or consistent way.

Line 430; although this was not measured in a structured method in this study.

This reviewer believes that structured observations play an important part in reducing bias and gathering reliable and subjective data on environmental conditions and behavioural practices (not least because problematic behaviours such as lack of handwashing with soap after toilet use, or open defecation, tend to be underreported and therefore suffer from risk of bias), and would have benefited the robustness of this study. Additionally, it is unclear whether efforts were made to utilise local government data on overall rates of access to improved drinking water and sanitation facilities, and levels of open defecation in the study areas.
We agree that structured observations and secondary data would have benefitted this study by collecting reliable data. Nevertheless, both methods were not utilised in this study due to limited time and funding
- The population is clearly described and appropriate.
- It is not possible to comment on the sample size since the calculation has not been provided. This has done, seen line 198-202
- As far as I can tell, the statistical analysis was correct, but this is not my area of expertise.
- Given that ethical approval was obtained and that an informed consent process was followed, there are no concerns. However, there are some contradictions in the description of the consent process, which I have detailed in my comments.
Reviewer #2: All appropriate
Reviewer #3: The objectives of the study have been clearly articulated by authors; however the study design has major limitations in being able to address these objectives. There are some major concerns related to the link between the objectives and the methods, including:
1.      As a cross-sectional study, the authors can only look at the association between STH prevalence or intensity at the time of measurement and the risk factors in the three counties that have been chosen. Mention of a ‘slow decline’ is misleading as the study does not look at any change in prevalence over time. By design, authors themselves have chosen these three counties for having persistent higher prevalence (but no mention is made of measurements over the seven rounds).

We are agreeing that in this paper we did not demonstrate the slow decline of STH, but rather we were interested in showing the factors contributing to slow decline. We have however explained what we mean by slow decline owing to past studies by the SBDP in the same schools and counties. Please see line 110-111.
2.      Related to the investigation into the three specific counties, without any comparison group(s), we do not know if the risk factors for STH infection are different than in other STH-endemic areas. The risk factors that have been found are primarily known risk factors related to WASH. A comparison with other areas or some consideration for a control area or group would have strengthened the findings.

We agree that a comparison study would have strengthened the findings of this study. However, the way we selected the schools, the study was not comparing the schools with low prevalence verses high prevalence but to understand why the six schools had maintained high to moderate prevalence of STH despite seven years of deworming (slow decline). See table 1.
3.      No cluster analysis was undertaken to account for potential clustering in schools or counties/subcounties. We have included how clustering was considered during analysis as shown in line; 267
4.      Regarding the age group, it is unclear why all grades from 1 to 7 were included as STH prevalence can be low in early childhood. Younger children have also benefited from few to no rounds of MDA already (unless they participated in Pre-SAC MDA. This is not clear). It would have been better to focus on the older grades with higher STH prevalence. As is, any subgroup analysis by age cannot be conducted due to low sample sizes.

Children in grades 1 to 7 were included in the study because they had participated in the previous mass drug administration (MDA). This gave a good representation of the whole school.
5.      The main indicator for analysis, results and discussion should be based on individual species intensity rather than prevalence (due to the impact of MDA on reduction in intensity and associated morbidity). Also, combining Ascaris, Trichuris and hookworm into one indicator for prevalence (any STH) may be warranted, but having one mean indicator for intensity for all STH species does not seem like a valid approach (as intensity categories have different meanings for the different species). Based on the results, it seems that Ascaris infection is driving the mean numbers.

Please refer to table 1 which has given intensity for each STH species.
6.      There is no discussion on any potential attempt to measure compliance, if these specific children were involved in previous rounds of MDA (unlikely the younger ones have had much opportunity), percentage of school children attending school, how many non-enrolled children were targeted in the interventions and study, if MDA was a standalone intervention or accompanied other interventions or health education (which may increase uptake). These details may provide more insight into if and why the intervention may not yet have achieved the desired results.

This study did not measure compliance and other interventions for the MDA among the SAC which has been stated as a study limitation. The study involved enrolled children attending school only.
7.      Authors state that the questionnaire was administered to all SAC (line 195). It is unclear how children could accurately respond to many of the questions. Questionnaires should have been administered to parents to get more accurate responses.

The questionnaire was designed to be administered to SAC who were the key target population for the study. During administration of the questionnaire, the SAC were assisted by their parents/guardians.
Some additional comments:
1.      Timeline: it is not clear when the study took place and if results are still current. Were results impacted at all by the pandemic (when coverage decreased in some areas)? The only place timeline is mentioned seems to be related to the timing of the MDA (2012-2018) and related to the data used to inform selection of sites (2017).

The study was conducted in in March 2019 as indicated in line 156.
2.      Sample size: no indication of sample size calculation and methodology for qualitative study. The sample size calculation has been included. See line 199-206.

The methodology for qualitative arm of the study is stated in line 245-251.
-Does the analysis presented match the analysis plan?</br>
-Are the results clearly and completely presented?</br>
-Are the figures (Tables, Images) of sufficient quality for clarity?</br></br>
Reviewer #1: The ability to interpret the results suffers from the same issues highlighted in the methods section. There seems to be confusion throughout this section between various identified factors, and the actual risk of STH infection. For example:
-       Toilet cleanliness in schools is mentioned – this is unlikely to be a direct cause of transmission; however, dirty toilets may cause users to prefer open defecation, which in turn increases environmental contamination. This has been explained in line 438-439.
-       The association between lack of handwashing with soap after toilet use and STH infection is mentioned – but as previously noted is more likely to be a proxy indicators of overall handwashing practices than a direct risk factor
-       The relationship between poverty indicators (such as not owning a sofa set) and STH infection is not adequately explained or interpreted Thanks for pointing this, we have incorporated and expounded on the explanation as suggested. See line 524-526
-       The association between T. trichiura infection with geographical location and source of drinking water is not explained. This is explained for all STH as shown in line 491-495
As noted in the comments on the methods, a clear conceptual framework of STH transmission and the various links with water supply, sanitation and specific hygiene behaviours would have, in my view, made it easier to organise and interpret the results.

A conceptual frame work has been provided. See line 148
Reviewer #2: appropriate
Reviewer #3: The analyses presented do match the analysis plan; however there are major limitations to presentation and interpretation of the results based on the same limitations previously outlined in the methodology.
For figures:
1.      Ideally there should be a figure/diagram to show the flow of participants. Rather than the current Fig. 1, this would be better expressed as a figure showing the actual study/participant flow (in the results section) and not the proposed flow/methodology (as part of the methods. This can still be included in written form). This has been explained in the method section see line 207-211.
<b>Conclusions</b></br></br>
-Are the conclusions supported by the data presented?</br>
-Are the limitations of analysis clearly described?</br>
-Do the authors discuss how these data can be helpful to advance our understanding of the topic under study?</br>
-Is public health relevance addressed?</br></br>
Reviewer #1: -  At the start of the discussion, the risk of reinfection could be expanded upon by referencing the systematic review: Jia TW, Melville S, Utzinger J, King CH, Zhou XN. Soil-transmitted helminth reinfection after drug treatment: a systematic review and meta-analysis. PLoS Negl Trop Dis. 2012;6(5):e1621. doi: 10.1371/journal.pntd.0001621. Epub 2012 May 8. PMID: 22590656; PMCID: PMC3348161. This reference has been added see line 435.
-       The issue of confusing toilet conditions/cleanliness with the risk of infection emerges in the discussion several times – again, it would be important to explain the relationship between this and the risk of infection: for instance, dirty toilets can lead to people preferring to practice open defecation; or poor state of repair of the infrastructure can result in faecal sludge leaking into the environment. This has been addressed in line 532-533.
-       Study limitations should be broadened to include several additional aspects:
o       Lack of structured observations
o       Lack of triangulation with other information sources/ secondary data
o       Lack of examination of all transmission pathways such as food crop contamination, testing overall environmental contamination, measuring other conducive aspects such as soil humidity, temperature etc.
While there are valid reasons for not including this aspects (mainly cost implications presumably), they should be set out as limitations nonetheless.

These has been improved in line 549-553.
-       It is unclear what the suggestion of using CLTS (row 448) is based upon. CLTS is designed to stop open defecation, but on its own does not necessarily lead to the construction of improved facilities that safely separate humans from faeces, and that are more likely to be maintained and sustained over time. It is only the starting point and should not be treated as a silver bullet (see WHO Guidelines on Sanitation and Health, Chapter 5). Approaches to improving overall sustained access and use should be based on the local context.

This has been revised see line 504-507.
-       The explanation of the link between lack of handwashing with soap after defecation (rows 448-451) is not biologically plausible, given the need for soil maturation of the eggs for faecal-oral transmission of ascaris and trichuris. As noted, this association is not likely to be indicative of causality, but rather a proxy of overall handwashing practices. This has been addressed see line 504-511.
-       While I agree with the final conclusion, I would suggest a deeper understanding is still needed of the causes for persistently high prevalence in the study area, in order to inform the integrated control measures advocated by the authors.This statement has been added on line 560-561.
Reviewer #2: appropriate
Reviewer #3: Due to the limitations listed above in the study design, the conclusions are limited and not supported directly by the data as presented. The potential applicability and generalizability of the conclusions are also limited. Authors have stated very few limitations of the study. The limitations have been added as suggested in line 549-553.
<b>Editorial and Data Presentation Modifications?</b></br><br/>
Use  this section for editorial suggestions as well as relatively minor modifications of existing data that would enhance clarity. If the only modifications needed are minor and/or editorial, you may wish to recommend “Minor Revision” or “Accept”.
Reviewer #1: Revisions suggested by row number:
60: Delete 'Helminth' from start of sentence (redundant) Helminth has been deleted
61: Targeted by the World Health Organization. Define EPHP. EPHP has been defined in line 62
63: the STHs now include strongyloides. Either include here or explain that the paper focuses only on the other three. Line 65; Strongyloides stercoralis has been included as STHs.
71: Ensure consistency in reference to road map targets (morbidity control mentioned here while eariler, the reference was to EPHP). This has been corrected see line 80
73-74: sentence unclear. The sentence has been revised line 80-82.
76: Please explain the predictive maps being referred Please see line 86-88.
81: mention prevalence threshold for MDAs. See line 101-105. Please note that there is no prevalence threshold for MDA. Treatment coverage (MDA) should be above 75%. Yearly treatment with coverage MDA (above75%) should lower the infection prevalence to a level were STH is no longer a public health problem (less than 1%).
86: please clarify how many of the 66 sub-counties mentioned in the previous paragraph are covered. The M & E reports gives number of schools, subcounties and counties covered during routine monitoring and evaluation of the school based deworming programme. The link is (<https://doi.org/10.1186/s13071-019-3322-1> )
90: replace "infection" with "prevalence" Infection has been replaced with prevalence see line 105
90-91: mention that 7 rounds of MDA took place during 2012-2018. Please mention the coverage level achieved by those MDAs.See line 103-105.Approximately over five million school children were reached with over 75% coverage in seven rounds of MDA that took place during 2012-2018 and the country-wide prevalence
92-93: please swap this sentence with the previous sentence. The two sentences have been swapped see line 101-105.
96-97: unclear whether the prevalence levels are for all STH. If there is a breakdown of the species, please provide this in table form. It is corrected please see line 101
102: the strategy period was 2015-2020, not 2015-2022. It has now been succeeded by a renewed strategy 2021-2030 (same timeframe as the NTD road map). Please provide a brief summary of the strategic objectives. This has been addressed, see line 119.
104: The sentence about the WASH and NTDs sectors is unclear. You could improve this by specifying that the opportunities described can be realised when there is collaboration between those two sectors, including raising awareness, using data for decision making, increasing the evidence base for action, and joint planning.

This sentence has been revised in line 127-131
107: "Primary prevention strategies" are mentioned but not clearly set out. Please see line 132-133
109-110: setting targets is only one aspect - ultimately, what is needed are interventions to improve access to water supply and sanitation services - and improvement of other preventive practices, noting that STH infection can result from contaminated crops as well.

This sentence has been revised in line 135-137. Ultimately, interventions to improve access to water supply and sanitation services and improvement of other preventive practices, noting that STH infection can result from contaminated crops as well
119 onwards (study area section): Please be consistent in terms of the information provided for each of the counties. (e.g. number of households and population density). A map would be helpful.  We are sorry we do not have a map for now.
127: what proportion of the population practices pastoralism? This has a very important bearing on aspects such as open defecation. Please note that we did not collect this information.
141: replace "where" with "were" this has been done in line 176
142: What type of M&E report? Coverage? Impact? Prevalence? This are impact evaluation report

Replace "where" with "were". This has been corrected in line 178
149: Who participated in the community meetings? Line 184; Community meetings with parents/guardians of the selected SAC
173: Please include age range. The age range has been included in line 203-204 .

185: Please include the name of the hospital/s. Line 219-221;
190: please mention the number of technologists. Please write Ministry of Health in full. This has been done in line 226; three trained medical laboratory technologists from Ministry of Health (MOH)
194: Please provide detail on questionnaire pretesting. This details have been provided, see line 232-234
205: Please summarise what information was gathered through FGDs, or at least the purpose of the FGDs. This has been done see line 245-251
216-217: Please mention that consent was sometimes obtained from parents/guardians rather than directly from child participants (mentioned earlier). Line 259-261; Written informed consent was sought from parents/guardians of SAC while the children gave assent for the quantitative arms of the study.
218: Sentence beginning with "assent" redundant? That sentence has been deleted
246: replace "and" with "from the"? Line 292; the word has been replaced
300: "poor WASH conditions both at school and at home" should be broken down: what is the access and coverage of improved household and school toilets? what is the access and coverage of improved drinking water supplies (improved)? And if available: handwashing facilities with water and soap. We would kindly wish to inform the reviewer that additional detailed information of the toilet/latrine other than what is presented in the tables were not measured in the study.
316: the word "ignorance" is perhaps not the right one to use here, as it is value laden. Consider referring to lack of awareness of or capacity to construct and maintain basic toilets.

This has been corrected in line 362; lack of awareness to construct and maintain basic toilets
346: Perhaps refer to the broader community rather than just the parents are reservoirs. Additionally, the main reservoir of risk of infection is more likely the environment itself given egg resilience. This has been corrected in line 392-393; STH since the community members and the environment if contaminated with faecal matters could be reservoirs
354: Frequency of annual MDA - you may wish to refer to the WHO or national treatment guidelines. Line 404-406; The current recommended WHO treatment schedule is once or twice annual administration which is usually determined by the initial prevalence of infection with any STH
366: Consider referring to health promotion rather than health education. This is a more up to date view of how behaviour change may be achieved. Line 414; has been changed to health promotion
390: reconsider the use of "ignorance" as mentioned above. Line 438; lack of awareness to construct and maintain basic toilets
408: you may wish to refer to the need for alternative treatment strategies to reduce the risk of resistance. This has been addressed in line 459
416-417: please clarify that eggs remain viable for longer in warm, moist soil conditions. Line 468-470; It is worth to note that warm weather in this regions could be a conducive habitat for this parasitic infection considering that STH eggs remain viable for longer in warm, moist soil conditions
418: replace "where" with "were" This has been replaced in line 471
442: Please clarify what is meant by "farming activities" - is this a reference to contamination of crops with faecal matter? This has been done in line 498 -499;
459-461: the last sentence of the paragraph is misplaced. The sentence has been aligned to support the previous one. Line 522
487-488: the final sentence is unclear. The sentence has since been deleted.
Reviewer #2: (No Response)
Reviewer #3: The article would benefit from overall editorial review to improve language, clarity and conciseness.
<b>Summary and General Comments</b></br></br>
Use  this section to provide overall comments, discuss strengths/weaknesses of the study, novelty, significance, general execution and scholarship. You may also include additional comments for the author, including concerns about dual publication, research ethics, or publication ethics. If requesting major revision, please articulate the new experiments that are needed.
Reviewer #1: This is an important study. It provides valuable impetus to addressing the weaknesses of MDAs as a disease elimination strategy as opposed to disease control imperatives, and highlights the need for increased collaboration between the WASH and NTDs sectors. However, it should be significantly strengthened as suggested in the detailed comments to ensure that it provides a clearer and more robust interpretation of the current situation in Kenya; define more specifically the role of water, sanitation and hygiene interventions; and set out a clear way forward in achieving both national and global NTD targets.
The most fundamental of my concerns refers to the lack of a conceptual framework that sets out clearly the transmission pathway of STH; as noted, the study has not addressed all pathways, and has not explored in depth the relationship of water, sanitation and hygiene aspects with each pathway. This inevitably leads to a narrow view of the measures needed to achieve elimination, which in turn can undermine efforts to improve cross sectoral collaboration and funding. While the study can of course not be amended retrospectively, I believe that presenting the conceptual framework and using this to interpret the results as well as set out the agenda for further research would greatly improve the paper. A conceptual frame work has been added see line 148.
Reviewer #2: Reviewer’s Comments
MS Ref.No.: PNTD-D-21-01677
Reviewer: Professor Berhanu Erko
Title: Understanding factors responsible for the slow decline of soil-transmitted helminths following seven rounds of annual mass drug administration (2012 – 2018) among school age children in endemic counties of Kenya: A mixed method study
General Comments: The paper reports the factors that could be influencing the slow decline of STH infections among school children after seven rounds of MDA in endemic counties of Kenya. It has addressed important problem and has also recommended implementation of integrated control efforts of STH by inclusion of WASH programmes, health education for awareness creation on behavior change communication. The paper is suitable for publication in PLOS Neglected Tropical Diseases after the authors have addressed the following specific comments:-
Specific comments
¬       Title:-line 1: It is suggested to replace “soil-transmitted helminths” with “soil-transmitted helminthiasis” in the title as it is more appropriate to refer to the infection rather than the parasite in this regard. This has been done in line 2; **helminthiasis**
¬       The phrase “school age children” in the title and other parts of the document:- The study participants were selected from enrolled primary schools and were, therefore, “school children”.  On the other hand, the authors referred to the study participants as  “school age children (SAC)” when they have appropriately defined school age children as “both enrolled and  non-enrolled” in lines 86 &87. Hence, it is appropriate to refer to the study participants as “school children” rather than “school age children (SAC)” throughout the paper. We agree that the National School-Based Deworming Programme (NSBDP) had been targeting school age children (SAC) both enrolled and non-enrolled. Nevertheless, for the purposes of this study, only enrolled SAC participated in the study
¬       Page 2, lines 34 & 35 need re-writing for clarity. Line 34-36 has been clarified
¬       Page 7, lines 141 & 142: replace “where” with “were” this has been done in line 176
¬       Page 7, lines 143: replace “Each study participants were” with “Each study participant was” This has been corrected in line 178; Each study participant was
¬       Line 163: Better to rewrite  “Fig 1: Study profile” as “Figure 3:  Study profile” in the caption and as Fig. in the in-text citation (lines 121 & 279). This has been changed see line 188 & 190
¬       Lines 291 &292: the statement that reads “This was echoed by the parents/guardians of the SAC as per the themes below;” needs qualification as there are many things below. To which things below the phrase refers? This also holds true for lines 340 & 341. The statement has been re-written see line 387-388
¬       The statement in lines 340 & 341 is vague and need re-writing for clarity. The statement has been re-written see line 387-388.
¬       Page 19, lines 398 – 400: Differences in mode of transmission cannot be a good explanation for differences in prevalence of the three STHs in this case as long as the children are in the same environment. In case of hookworms, only wearing shoes can make a difference. This has been addressed see line 447-452.
¬       Page 21, line 464: Better not to provide statistical test results in the discussion section. The statistical test results have been deleted
¬       Page 22, line 482:re-write the statement that reads “…egg count in an area with low prevalence especially after several rounds…” as “…eggs in an area with low intensity of infection especially after several rounds…” This has been corrected in line 544
¬       Line 507: replace “Ethics approval and consent to participate” simply with “Ethical approval” or “Ethics statement” because consent to participate is an aspect of ethical approval. This has been corrected in line 574; Ethics statement
¬       Lines 512 - 514: Delete the sentence that reads “Verbal consent was also obtained from the study participants by explaining the study purpose, confidentiality, and the procedures to be followed”, because the study participants were children for whom proxy consent is sought from parents or guardians. The sentence has been deleted
¬       Lines 514 - 515: Provide age range as “13 to _” instead of above 13 years. This has been addressed see line 579-580.
Reviewer #3: Overall, the authors have conducted a study that has the potential for both local and international relevance as the impact of MDA after several rounds is monitored and adjusted accordingly (scaled up or scaled down). Knowing why there is persistent infection in some areas and not others, and incorporating community input and feedback into the MDA process are important goals. However, as is, the authors are unable to elucidate the reasons for continued STH infection in these areas – only what the current risk factors are. The qualitative results are also interesting but may not translate into direct changes to the interventions. Factors such as compliance, accompanying interventions, logistical details related to the MDA, etc. are needed to better understand the potential shortcomings of these interventions.

We agree that factors like compliance, logistical details and accompanying interventions related to the seven year MDA are needed to better understand the potential shortcomings of the intervention. Nevertheless, due to limited funding and time this study did not investigate these factors which have been presented as study limitations see line 542.

We look forward to your consideration. Thank you.

Janet M. Masaku; Corresponding author
