# Supplementary material for: Understanding factors responsible for the slow decline of soil-transmitted helminthiasis following seven rounds of annual mass drug administration (2012–2018) among school children in endemic counties of Kenya: A mixed method study
Source: PLoS Negl Trop Dis. 2023 May 4;17(5):e0011310. doi: 10.1371/journal.pntd.0011310 (PMC10187929; doi:10.1371/journal.pntd.0011310)
Supplement: Attachment — Submitted filename: Comment_PNTD-D-21-01677R1.docx [file pntd.0011310.s002.docx]

**Comment on the revised version (PNTD-D-21-01677R1)**

**Title:** Understanding factors responsible for the slow decline of soil-transmitted helminthiasis following seven rounds of annual mass drug administration (2012 – 2018)

among school age children in endemic counties of Kenya: A mixed method study

The authors have addressed more or less my previous comments but have not considered my comments on school age children (SAC). According to WHO definition, SAC includes both enrolled and non-enrolled children. On the other hand, the study participants of the reported work were enrolled school children. I recommend acceptance of the work for publication after considering this comment.
