# Supplementary material for: Understanding factors responsible for the slow decline of soil-transmitted helminthiasis following seven rounds of annual mass drug administration (2012–2018) among school children in endemic counties of Kenya: A mixed method study
Source: PLoS Negl Trop Dis. 2023 May 4;17(5):e0011310. doi: 10.1371/journal.pntd.0011310 (PMC10187929; doi:10.1371/journal.pntd.0011310)
Supplement: Attachment — Submitted filename: Responses to reviewers comments 2.docx .docx [file pntd.0011310.s003.docx]

The Chief Editor,

PLOS Neglected Tropical Diseases,

Dear Sir/Madam,

**REFERENCE: SUBMISSION OF A REVISED MANUSCRIPT**

We are hereby, submitting a revised manuscript titled **“Understanding factors responsible for the slow decline of soil-transmitted helminths following seven rounds of annual mass drug administration (2012 – 2018) among school children in endemic counties of Kenya: A mixed method study”**, for your consideration for publication in your esteemed journal.

We have acted on the reviewer’s comments as listed below;

Reviewer #1: - A conceptual framework was added following the previous review. However, it does not help address the transmission pathway of STH, which is what was missing in order to be able to guide the study and interpret the results. For instance, the framework does not explain that helminth eggs must mature in soil before they can infect a new host, and does not explain how different WASH aspects affect transmission. As it stands, it is confusing and should be removed unless the authors can provide an alternative.

The conceptual frame work has been removed as suggested by the reviewer.

- While the authors have added some reference to the contents of the WHO Guidelines on Sanitation and Health, the main point raised in earlier comments was that the release of excreta (and therefore helminth eggs) can occur at every stage of the sanitation service chain, not just through open defecation and whether or not a toilet is present

This has been addressed as stated in line 128-132; In addition, WHO guidelines recommend sanitation systems to be designed and managed safely to protect human health from microbial hazards caused by the release of human excreta (and therefore helminth eggs) which can occur at every stage of sanitation of the service chain and consequently adverse health outcomes.

- The authors seem not to have address the issue of the association between handwashing after defecation and transmission and this problem is repeated throughout the paper. To explain further - handwashing after defecating is not likely to reduce transmission of STH since the eggs transmitted through this pathway will not have matured in the soil in order to become infective. Handwashing after defecating should therefore be treated as a proxy of handwashing more generally, includling before eating - which is more likely to be the relevant transmission pathway. This is alluded to in the discussion (row 508 onwards) but not sufficiently covered or emphasised in the rest of the paper incuding the conclusion.

This has been addressed in 512-514; Lack of handwashing with soap and water after defecation was a proxy indicator of broader personal hygiene/handwashing practices, and therefore a possible pathway towards STH infection.

In addition, in the conclusion it has been corrected.

- The constraints faced by the researchers, which meant that structured observations could not be undertaken, are understood and cannot be addressed at this stage. However, these should be more thoroughly descrived in the study limitations.

This has been corrected as stated in line 557-559; In addition, lack of structured observations of water supply, sanitation infrastructure and associated behaviours was a major limitation to the study due to limited funding which could have improved the study outcomes.

Reviewer #2: (No Response)

Reviewer #3: Improved from previous version. Responded to reviewers' comments.

--------------------

<b>Results</b></br></br>
-Does the analysis presented match the analysis plan?</br>
-Are the results clearly and completely presented?</br>
-Are the figures (Tables, Images) of sufficient quality for clarity?</br></br>

Reviewer #1: - The issue of the association of handwashing after defecation and infection comes up again here and the authors have not addressed earlier comments that this should not be treated as a direct relationship. It is a proxy of broader hygiene/handwashing practices, in the same way that lacking a sofa set is a proxy indicator of poverty (as the authors themselves have noted). As pointed out, the lack of an appropriate conceptual framework makes the analysis harder to organise and interpret.

This has been addressed in line 512-513; Lack of handwashing with soap and water after defecation was a proxy indicator of broader personal hygiene/handwashing practices, and therefore a possible pathway towards STH infection

Reviewer #2: (No Response)

Reviewer #3: Improved from previous version. Responded to reviewers' comments.

--------------------

<b>Conclusions</b></br></br>
-Are the conclusions supported by the data presented?</br>
-Are the limitations of analysis clearly described?</br>
-Do the authors discuss how these data can be helpful to advance our understanding of the topic under study?</br>
-Is public health relevance addressed?</br></br>

Reviewer #1: - The conclusions are weakened by the rest of the analysis as pointed out above. Since the association with handwashing after defecation is referred to without the necessary caveats pointed out above, and no secondary data was included to explain the overall sanitation conditions in the surveyed districts, it is very hard to draw firm conclusions except for "more research is needed".

This has been addressed in line 568-569; hence, more research is needed to understand the causes of persistent high prevalence of STH infection in the selected counties.

- The limitations should be more thoroughly explained since they can inform the design of the subsequent research the authors are calling for in the conclusions. For instance, "structured observations of water supply and sanitation infrastructure and associated behaviours", instead of just "structured observations".

This has been addressed in line 554-557; In addition, lack of structured observations of water supply, sanitation infrastructure and associated behaviors was a major limitation to the study due to limited funding which could have improved the study outcomes.

Reviewer #2: (No Response)

Reviewer #3: Improved from previous version. Responded to reviewers' comments.

--------------------

<b>Editorial and Data Presentation Modifications?</b></br><br/>
Use  this section for editorial suggestions as well as relatively minor modifications of existing data that would enhance clarity. If the only modifications needed are minor and/or editorial, you may wish to recommend “Minor Revision” or “Accept”.

Reviewer #1: - Reference to strongyloides has been added, but this is not addressed by the study, which needs to be noted.

This has been addressed on line 66; It is worth to note that *S. stercoralis* was not addressed in the current study.

- Prevalence thresholds for MDA should still be included - as far as I understand, MDA is recommended by WHO when the prevalence is over 20 percent.

This has been addressed in line – 112-119. It is expected that after seven years of annual MDA, the disease prevalence will be lowered to the point at which a reduction in the frequency of subsequent rounds of MDA can be considered. According to the WHO STH decision tree (18), the MDA regimen should be continued according to a set of endemicity classes defined by prevalence thresholds as follows: suspend MDA if STH prevalence is less than 2%; conduct MDA every 2 years if STH prevalence is between 2% and 10%; annually if between 10% and 20%; twice yearly if between 20% and 50%; and thrice yearly if greater than 50% (19).

- Health education - please apply the revision to "health promotion" in the rest of the paper.

This has been done.

Reviewer #2: (No Response)

Reviewer #3: (No Response)

--------------------

<b>Summary and General Comments</b></br></br>
Use  this section to provide overall comments, discuss strengths/weaknesses of the study, novelty, significance, general execution and scholarship. You may also include additional comments for the author, including concerns about dual publication, research ethics, or publication ethics. If requesting major revision, please articulate the new experiments that are needed.

Reviewer #1: This study raises important issues of interest to the scientific community, especially NTDs. It rightly points out that while poor WASH conditions persist, elimination of STH as a public health promblem, or indeed interruption of transmission, will not be possible.
Nonetheless, the authors should make every effort to not overstate the findings given the limitations to the study caused by (very legitimate) cost and time constraints. They should therefore carefully set these out as pointed above in order to lay out clear recommendations for future research.

This has been addressed in line 564-565; Hence, more research is needed to understand the causes of persistent high prevalence of STH infection in the selected counties. Hence, more research is needed to understand the causes of persistent high prevalence in the selected counties.

Reviewer #2: (No Response)

Reviewer #3: (No Response)

The authors have addressed more or less my previous comments but have not considered my comments on school age children (SAC). According to WHO definition, SAC includes both enrolled and non-enrolled children. On the other hand, the study participants of the reported work were enrolled school children. I recommend acceptance of the work for publication after considering this comment.

School age children (SAC) has been replaced with school children were necessary throughout the text.
