# Supplementary material for: Understanding factors responsible for the slow decline of soil-transmitted helminthiasis following seven rounds of annual mass drug administration (2012–2018) among school children in endemic counties of Kenya: A mixed method study
Source: PLoS Negl Trop Dis. 2023 May 4;17(5):e0011310. doi: 10.1371/journal.pntd.0011310 (PMC10187929; doi:10.1371/journal.pntd.0011310)
Supplement: Attachment — Submitted filename: PNTD-D-21-01677_R2 Recommendation.docx [file pntd.0011310.s004.docx]

**Understanding factors responsible for the slow decline of soil-transmitted helminthiasis following seven rounds of annual mass drug administration (2012 – 2018) among school children in endemic counties of Kenya: A mixed method study (Ms No.: PNTD-D-21-01677_R2)**

**Recommendation:** The authors have now addressed my comments. I recommend publication of the paper.
